# Text-mined dataset of inorganic materials synthesis recipes

**DOI:** 10.1038/s41597-019-0224-1

**Published:** 2019-10-15

**Authors:** Olga Kononova, Haoyan Huo, Tanjin He, Ziqin Rong, Tiago Botari, Wenhao Sun, Vahe Tshitoyan, Gerbrand Ceder

**Affiliations:** 1 Department of Materials Science and Engineering, University of California, Berkeley, CA 94720 USA; 2 Materials Sciences Division, Lawrence Berkeley National Laboratory, Berkeley, CA 94720 USA; 3 Present Address: Institute of Mathematics and Computer Sciences, University of São Paulo, São Carlos, SP Brazil; 4 Present Address: Google LLC, Mountain View, CA USA

**Keywords:** Computational methods, Solid-phase synthesis

## Abstract

Materials discovery has become significantly facilitated and accelerated by high-throughput *ab-initio* computations. This ability to rapidly design interesting novel compounds has displaced the materials innovation bottleneck to the development of synthesis routes for the desired material. As there is no a fundamental theory for materials synthesis, one might attempt a data-driven approach for predicting inorganic materials synthesis, but this is impeded by the lack of a comprehensive database containing synthesis processes. To overcome this limitation, we have generated a dataset of “codified recipes” for solid-state synthesis automatically extracted from scientific publications. The dataset consists of 19,488 synthesis entries retrieved from 53,538 solid-state synthesis paragraphs by using text mining and natural language processing approaches. Every entry contains information about target material, starting compounds, operations used and their conditions, as well as the balanced chemical equation of the synthesis reaction. The dataset is publicly available and can be used for data mining of various aspects of inorganic materials synthesis.

## Abstract (summary)

**Table:** 

Measurement(s)	solid-state synthesis data
Technology Type(s)	natural language processing

Machine-accessible metadata file describing the reported data: 10.6084/m9.figshare.9906608

## Background & Summary

The number of big-data-driven projects for materials discovery has been boosted significantly in the last decades due to Materials Genome Initiative efforts^1^ and growth of computational tools^2–6^. Building and maintaining of large-volume databases has become a crucial step to provide scientific data for mining and modeling. Widely used materials databases, such as the Inorganic Crystal Structure Database (ICSD)^7,8^, NIST Web-book^9^, the Pauling File and its subsets^10,11^, have been manually constructed and curated over decades and store experimentally obtained data for thousands of inorganic materials. Combining high-throughput computations with database infrastructure has led to the establishment of large-scale databases with *ab initio*-calculated materials structures and properties^12–16^.

At the same time, scientific publications have accumulated an enormous amount of information about materials, but the data is presented in unstructured and arbitrary form which significantly obstructs its use in data-driven research^17^. Early approaches to text-mining of materials data have been implemented by manual extraction from a limited amount of articles^18^, and lab notebooks^19^. Development of text mining and natural language processing (NLP) approaches have made it possible to implement various automated methodologies for converting scientific text into structured data collections^20,21^. Among the most widely used NLP toolkits for chemical text processing and information extraction are ChemDataExtractor^22^, OSCAR4^23^, ChemicalTagger^24^ and others^17,25^.

Most of the existing data extraction and mining developments have been applied to establish and predict structure-property-function relationships for materials^26–29^. Only recently effort has been spent to create collections of materials synthesis data and using them to predict materials synthesis routes^19,30^. Kim *et al*. created publicly available dataset of inorganic synthesis parameters for 30 different oxides systems extracted from literature^20^. They used their data to provide guidelines for titania nanotubes synthesis^30^. AI-guided synthesis predictions for organic molecules have already been applied successfully^31–33^, as organic reaction data is more widely presented in well-structured and machine readable format^34,35^.

**Fig. 1:**
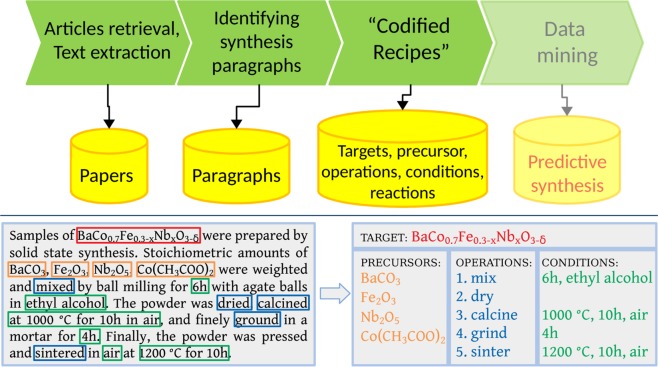
Schematic representation of synthesis “recipes” extraction pipeline. *Top panel:* The pipeline starts with retrieval of HTML content from major publishers which is then parsed into a raw text. Next, paragraphs describing synthesis are identified and classified according to synthesis type. Every paragraph is then processed to extract synthesis “recipe”, i.e. materials, operations and conditions. The output is stored in a database for further data mining. *Bottom panel:* Example of processing a synthesis paragraph into a “recipe”. The key component of “recipe”, such as target and starting materials, synthesis steps and their conditions are found and extracted from the paragraph by different text mining algorithms (see Methods).

In this work, we provide fully auto-generated open-source dataset of 19,744 chemical reactions retrieved from 53,538 solid-state synthesis paragraphs. The data are collected using an automated extraction pipeline (Fig. 1) which converts unstructured scientific paragraphs describing inorganic materials synthesis into so-called “codified recipe” of synthesis. The pipeline utilizes a variety of text mining and NLP approaches to find information about target materials, starting compounds, synthesis steps and conditions in the text, and to process them into chemical equation. The dataset is publicly available in JSON format. Digitizing and systemizing the large corpus of existing solid-state chemistry literature enables us to make a first step toward development of data-driven approaches for understanding inorganic materials synthesis and synthesizability.

## Methods

### Content acquisition

Scientific publications used in this work are journal articles published by Springer, Wiley, Elsevier, the Royal Society of Chemistry, the Electrochemical Society, and the American Chemical Society, from which we received permissions to download large amounts of web-content. For each publisher, we manually identified all materials science related journals available for download. A web-scraping engine was built using the *scrapy* (https://scrapy.org/) toolkit. Since the full-text articles published before 2000’s are mostly in PDF format, which complicates their parsing, we chose to process only papers in HTML/XML format published after the year 2000. The downloaded content includes the text of the article as well as its metadata such as journal name, article title, article abstract, authors, etc. All data was stored in a document-oriented database implemented using a MongoDB (www.mongodb.com) database instance. Because downloaded articles contain irrelevant markups, we developed a customized library for parsing article markup strings into text paragraphs while keeping the structure of paper and section headings.

### Paragraphs classification

To find paragraphs on solid-state synthesis, we used a two-step paragraph classification approach described elsewhere^36^ which consists of an unsupervised algorithm to cluster common keywords in experimental paragraphs into “topics” and generate a probabilistic topic assignment for each paragraph, followed by a random forest (RF) classifier trained on annotated paragraphs. The outsome of the RF is a classification of the synthesis methodology in a paragraph as either solid-state synthesis, hydrothermal synthesis, sol-gel precursor synthesis, or “none of the above”. The annotation set consisted of 1,000 paragraphs for each label.

### Synthesis recipe extraction

A typical synthesis procedure in the solid-state chemistry literature contains information about precursor and target materials, synthesis operations and operation conditions. These items comprise a materials synthesis “recipe” and were extracted from a synthesis paragraph as shown in Fig. 1. Our extraction pipeline consists of several algorithms which analyze a paragraph and identify information about materials (final products and starting precursors), synthesis steps performed, and conditions associated with those steps. Finally, target and starting materials as well as synthesis conditions are used to balance a chemical equation representing the synthesis reaction. The next sections provide details on each step of the pipeline.

#### Material entities recognition (MER)

To identify starting materials and final products mentioned in a synthesis paragraph, we implemented a bi-directional long-short term memory neural network with a conditional random field layer on top of it (BiLSTM-CRF)^37,38^ which is able to recognize the meaning of a word based on both the word itself and its context. Extraction was performed in two steps each executed by a different neural network: first we identified all materials entities presented in the paragraph; next we replaced each material with a keyword “<MAT>” and classified them as TARGET, PRECURSOR or OTHER material. Each word input for the BiLSTM-CRF was represented as the combination of a word-level embedding from a Word2Vec model^39^ trained on ∼33,000 solid-state synthesis paragraphs, and a character-level embedding from a character lookup table. The table was randomly initialized and then optimized during the training of the BiLSTM-CRF. As an additional feature in the word representation for the second neural network, we also included chemical information about each material, i.e. number of metal/metalloid elements and a flag indicating whether the material contains C, H and O elements only. This assisted in the differentiation of precursors and targets, as they tend to have different number of metal/metalloid elements and are generally not organic compounds in our dataset. We manually annotated 834 solid-state synthesis paragraphs from 750 papers by assigning each word token with the following tags: “material”, “target”, “precursor”, and “outside” (not a material entity). The annotated dataset was randomly split into training/validation/test sets with 500/100/150 papers in each set. The model parameters were iteratively optimized on the training set using early stopping regularization^40^ to minimize overfitting, and the model with best performance on the validation set was chosen.

#### Synthesis operations

We implemented an algorithm which combines neural network and sentence dependency tree analysis to identify key steps of solid-state synthesis given in the paragraph. The neural network was used to classify sentence tokens into 6 categories: NOT OPERATION, MIXING, HEATING, DRYING, SHAPING, QUENCHING, which are the main operations in solid-state synthesis. To create tokens features, we trained a Word2Vec model^39^ on ∼20,000 synthesis paragraphs using the Gensim library^41^. For the Word2Vec model training, the sentences of paragraphs were lemmatized, all the quantity tokens were replaced with a keyword <NUM>, and all the chemical formulas were replaced with keyword <CHEM>. We also used the SpaCy library^42^ to grammatically parse each sentence and obtain linguistic features of token such as token’s part of speech and its dependency to a root token. The annotated set consisted of 100 solid-state synthesis paragraphs (664 sentences) with manually assigned tokens labels. For training, validation and testing, the annotated set was split into a 70/10/20 fraction, respectively. Next, we used the dependency tree to assign MIXING operations as a SOLUTION MIXING if its lemma belongs to any solvent-based process (e.g ‘disperse’, ‘dilute’, ‘dissolve’, etc) or has a solution environment (e.g. ‘ethanol’, ‘water’, ‘alcohol’, etc.) in its sub-tree. This was differentiated from a MIXING operation which consists of grinding or milling in liquid environment, which was assigned the LIQUID GRINDING label.

#### Mixing and heating conditions

For every HEATING operation, we extracted the values or range of values for time, temperature, atmosphere corresponding to the operation, if they are mentioned in the same sentence. We applied a regular expression approach to find the values of temperature and time, and a keyword-search to find atmosphere. For any operation of type MIXING, we extracted corresponding mixing media and type of mixing device, if they are mentioned in the same sentence. For this, we used the list of materials labeled by MER as OTHER materials, as well as keyword-matching, to find potential device or media substances. The extracted attributes were assigned to both the heating and mixing by using dependency sub-tree analysis. Throughout the text, these attributes are referred as “conditions” of synthesis or operations.

#### Balancing equations

Every material entry was processed with a *Material Parser*, which converts the string representing the material into a chemical formula and splits it into elements and stoichiometries. Balanced reactions were obtained from parsed precursors and target materials by solving a system of linear equations. Variables of the linear equations represent molar amounts of materials involved in a reaction, and each equation asserts the conservation of a certain chemical element in the reaction. Besides precursor and target materials, we also included a set of “open” compounds (i.e. the compounds that can be released or absorbed during solid-state synthesis, such as O_2_, CO_2_, N_2_, etc.) which were inferred based on the compositions of precursor and target materials. Whenever a target material was synthesized with a “modifier”, i.e. doping, stabilizing, substituting elements, a note is assigned to the reaction: “target <target_name> with additives <element> via <precursor>”. To solve symbolic equations for materials with variable amounts of chemical elements, we used the Gaussian elimination routines in *SymPy*^43^.

### Dataset generation

We scraped a total of 4,204,170 papers, which contained 6,218,136 paragraphs in the experimental sections. The experimental sections were identified by using case-insensitive keyword matching in section headings (i.e. “experiment”, “synthesis”, “preparation” and their morphological derivations). Plain text paragraphs were segmented into sentences and tokenized into words using the ChemDataExtractor tokenizer^22^. After classification, 188,198 paragraphs were found to describe inorganic synthesis, such as solid-state, hydrothermal, sol-gel, co-precipitation syntheses, with 53,538 corresponding to solid-state synthesis. These 53,538 paragraphs and their corresponding abstracts were processed to extract materials, operations, conditions and balance chemical equation as described above.

## Data Records

The complete dataset of 19,488 solid-state synthesis reactions is provided as a single JSON file, and it is publicly available at 10.6084/m9.figshare.9722159.v3 ^44^. Each record corresponds to a single chemical reaction built from a paragraph describing inorganic material synthesis, and is represented as a JSON object in a top-level list. If a paragraph reports synthesis of several materials or a material with variable substituted elements, the corresponding reactions are split into separate data records. Aside from a balanced chemical equation, the metadata for each reaction include: DOI of the paper from which the reaction is extracted and a snippet of the corresponding synthesis paragraph (50 first and 50 last characters to facilitate its lookup), chemical information about target and precursor materials used in the reaction, operations and conditions for heating and mixing steps to synthesize the target material. The details of the data format are given in Table 1.

**Table 1:** Format of each data record: description, key label, data type.

YellowGreen Data description	Data Key Label	Data Type
DOI of the original paper	doi	*string*
Snippet of the raw text	paragraph_string	*string*
Chemical equation	reaction	Object (*dict*):
		- element_substitution:
		- left_side: *list* of Objects^a^
		- right_side: *list* of Objects^a^
Chemical equation in string format	reaction_string	*string*
Target material data	target	Object (*dict*):
		- material_string: *string*,
		- material_formula: *string*,
		- composition: *list* of Objects^b^,
		- additives: *list* of *strings*
		- elements_vars: {var: *list* of *strings*}
		- amounts_vars: {var: *list* of Objects^c^}
		- oxygen_deficiency: *boolean*
		- mp_id: *string*
List of target formulas obtained after variables substitution	targets_string	*list* of *strings*
Precursor materials data	precursors	*list* of Objects (See target)
Sequence of synthesis steps and corresponding conditions	operations	*list* of Objects (*dict*):
		- token: *string*,
		- type: *string*
		- conditions: Object
		–heating_temperature: *list* of Objects^d^
		–heating_time: *list* of Objects^d^,
		–heating_atmosphere: *list* of *strings*
		–mixing_device: *list* of *strings*
		–mixing_media: *list* of *strings*

^a^{amount: *float*, material: *string*}.

^b^{formula: *string*, elements: {element: amount of element}, amount: *string*}.

^c^{max_value: *float*, min_value: *float*, values: *list* of *floats*}.

^d^{max_value: *float*, min_value: *float*, values: *list* of *floats*, units: *string*}.

The chemical equation for the reaction is stored as a string as well as a list of pairs: chemical substance (material) and stoichiometric coefficient (amount). The reactants and products are listed in the left_side and right_side, respectively. If in the original paper the target compound was synthesized with variable substituted elements, the element used in the particular reaction is given in element_substitution.

The metadata for target and precursors used to construct and balance the chemical equation are represented by a data structure with the following properties:

- material_string: string of material as given in the original paragraph before being parsed into chemical composition.
- material_formula: chemical formula associated with the material (given originally or constructed empirically by parser).
- composition: chemical composition of the material derived from its formula. Aside from single compound materials, we found that a large portion of the materials (predominantly target materials) are composites, mixtures, solid solutions or alloys, written as sequence of ratio-compound pairs. Therefore, a chemical composition entity is represented by a list of dictionaries where each item is associated with a compound found in the materials formula. The ratio of each compound in the material is given in amount, its chemical composition (i.e. element and its fraction) is given in elements. If a material is one compound, the list has only one item and amount = 1.0. If a material is hydrate, the water is added into the composition list with the amount corresponding to the amount of water molecules (if specified).
- additives: list of additive elements (i.e. elements used for doping, stabilization, substitution) resolved from material string.
- elements_vars: lists all variable elements and their corresponding values found in the materials.
- amounts_vars: lists all variable elements ratios and their corresponding values found in the material formula. The values of each variable are given as a structure with values listing specific variable’s values, and max_value/min_value values if range is given in the paragraph.
- oxygen_deficiency: yes/no attribute which reflects if material was synthesized with unspecified oxygen stoichiometry.
- mp_id: ID of the lowest-energy polymorph entry in Materials Project database (materialsproject.org) if it is presented there.

To facilitate querying of the dataset, the targets_string field contains all target material formulas obtained by substituting amounts_vars in the material_formula.

The sequence of synthesis steps for the reaction (if specified in the paragraph) is listed as a data structure with the following fields: original token from the text (token), its type (type) as assigned by classification algorithm (see Methods) and conditions used at this step (conditions). If the synthesis step has type HEATING then temperature, time and atmosphere conditions are provided in the conditions attribute. Temperature and time are given as values if discrete values are given, or max_value/min_value if a range is given. If the synthesis step is of the MIXING type then the mixing device and mixing media are specified in the conditions attribute.

## Technical Validation

### Extraction accuracy

The overall extraction yield of the pipeline is 28%, meaning that out of 53,538 solid-state paragraphs, only 15,144 of them produce a balanced chemical reaction. As a test of the full extraction pipeline, we randomly pulled 100 paragraphs from the set of paragraphs classified as solid-state synthesis, and checked them against completeness of the extracted data. Out of the 100 paragraphs, we found 30 that did not contain a complete set of starting materials and final products, meaning that a human expert would not be able to reconstruct a reaction from these paragraphs. The remaining 70 paragraphs could potentially contribute to the dataset as they provide all information about starting materials and final products. Inspections of those 70 paragraphs showed that 42 potential reactions were not reconstructed due to an incomplete or overcomplete set of extracted precursor/target materials, or a failure to parse chemical composition, which makes it impossible to balance the reaction. The former loss originates from the lower re-call of the MER algorithm which we traded in for higher precision, while the parsing problem occurs due to complicated notation used for a materials entity.

Evaluation of the dataset records accuracy was performed by randomly pulling 100 entries and manually checking each extracted field against the original paragraph. The calculated precision, recall and F1-score for every attribute of the data entry is given in Table 2. Overall, we achieved a high accuracy in extraction of targets (precision 97%), precursors (F1-score 99%), operations (F1-score 90%) and balancing reactions (precision 95%). The lower accuracy of the heating conditions (F1-score < 90%) is mostly caused by the cases where the heating step is missed by the operations extraction algorithm. The retrieval of the mixing conditions show relatively poor accuracy with F1-score 65%. This is largely due to misidentification by MER of the device material or media substance used for mixing, as well as because those conditions are often not mentioned in same sentence as the mixing procedure.

**Table 2:** Performance of data extraction for dataset entries.

Data attribute	Precision	Recall	F1 score
**Materials**			
- targets	0.97	/	/
- precursors	0.99	0.99	0.99
Operations	0.86	0.95	0.90
**Heating conditions**			
- temperature	0.85	0.87	0.86
- time	0.90	0.88	0.89
- atmosphere	0.89	0.86	0.87
**Mixing conditions**			
- mixing media	0.62	0.66	0.64
- mixing device	0.82	0.55	0.66
Balanced reactions	0.95	/	/

This analysis leads us to a conclusion that at the chemistry level (correct precursors, targets, reactions), the accuracy of the dataset is 93%. When including all operations and their conditions, the accuracy of having all recipe items (chemistry, operations and attributes of the operations) extracted and assigned correctly is 51%, which is low due to low performance in extraction the mixing attributes. For many solid-state recipes, specifics of mixing the precursors is of less importance, so this extraction failure is less critical. When considering only correctness of the recipe without conditions for heating and mixing (i.e. chemistry, operations and reactions), the accuracy rises to 64%.

It is worth noting that for this dataset we aimed to achieve higher precision of the data extraction in expense of lower recall (i.e. better miss the data record, rather than provide the wrong one), therefore the extraction rate is low. Yet, constructing the balanced chemical equation sets up additional constraints on targets and precursors, and helps to reduce potential errors that may have been caused by composition parsing. This results in a skew of the metrics toward higher accuracy for identification of targets and precursors, as compared to operations.

### Dataset mining

In order to test the diversity of the entries representing the dataset, we first obtained a list of unique materials (targets and precursors) and reactions. The dataset contains 13,009 unique targets, 1,845 unique precursors and 16,290 unique reactions. The almost 10-fold lower variety of precursors compared to targets can be explained by the fact that in general researchers operate with a set of common well-established precursors. Table 3 represents the ten most frequent targets, precursors and reactions in the dataset. The target compounds neatly capture the types of materials most often studied in the last two decades via solid-state synthesis. These are lithium ion battery cathode materials (LiFePO_4_, LiMn_2_O_4_ and LiNi_0.5_Mn_1.5_O_4_), as well as perovskites for multiferrorics, LEDs and CMOS applications (BaTiO_3_, BiFeO_3_, SrTiO_3_, Y_3_Al_5_O_12_). It is possible that this “top-ten” materials list is biased by the set of publishers that gave us permission to access their scientific corpus. For example, The American Physical Society was not included and may have brought other compounds to the list.

**Table 3:** Ten most common targets, precursors and reactions present in the dataset.

Targets	Precursors	Reactions
LiFePO_4_	TiO_2_	BaCO_3_ + TiO_2_ = BaTiO_3_ + CO_2_
LiMn_2_O_4_	SrCO_3_	3CuO + 4TiO_2_ + CaCO_3_ = CaCu_3_Ti_4_O_12_ + CO_2_
BaTiO_3_	BaCO_3_	0.5Bi_2_O_3_ + 0.5Fe_2_O_3_ = BiFeO_3_
BiFeO_3_	La_2_O_3_	SrCO_3_ + TiO_2_ = SrTiO_3_ + CO_2_
CaCu_3_Ti_4_O_12_	CaCO_3_	2Li_2_CO_3_ + 5TiO_2_ = Li_4_Ti_5_O_12_ + 2CO_2_
SrTiO_3_	Bi_2_O_3_	TiO_2_ + CaCO_3_ = CaTiO_3_ + CO_2_
Li_4_Ti_5_O_12_	Fe_2_O_3_	Nb_2_O_5_ + ZnO = ZnNb_2_O_6_
Y_3_Al_5_O_12_	Nb_2_O_5_	6Fe2O3 + BaCO3 = BaFe12O19 + CO2
CaTiO_3_	Li_2_CO_3_	Li_2_CO_3_ + TiO_2_ = Li_2_TiO_3_ + CO_2_
LiNi_0.5_Mn_1.5_O_4_	Na_2_CO_3_	0.5Li_2_CO_3_ + 0.333Co_3_O_4_ + 0.083O_2_ = LiCoO_2_ + 0.5CO_2_

**Fig. 2:**
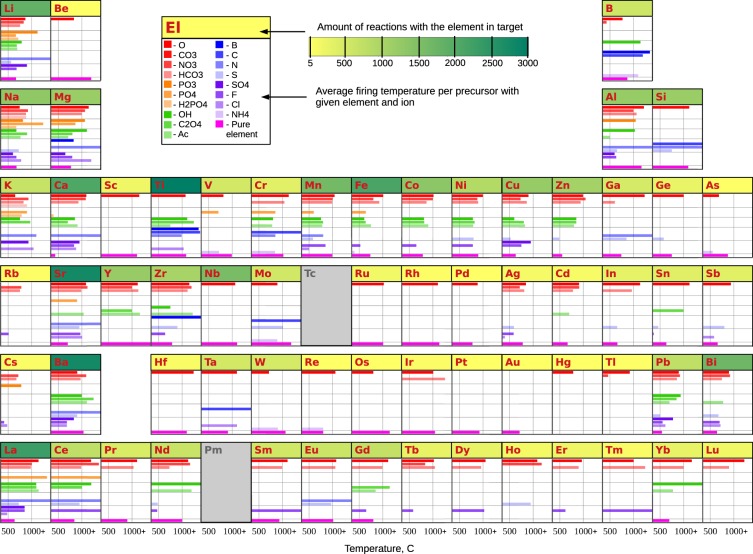
Map of chemical space covered by the dataset. For each element, the frame colored in a yellow-to-green gradient represents the total amount of reactions that produce a target compound containing the element. The bar graph below each element shows the list of ions paired with the element in precursor compounds. The length of the bar corresponds to the firing temperature averaged over all the reactions using the given precursor (i.e. element + counter-ion). The elements occurring in five and less targets are faded in grey. “Ac” stands for acetate radical CH_3_COO^−^ in the compound formula.

Next, we evaluate the chemical space covered by the dataset. For each chemical element, we computed the amount of the reactions which include the given element in the target. The results are mapped in Fig. 2 in the yellow-to-green gradient frame at the top of each element box. The database is dominated by target materials containing Ti, Sr, Ba, La, Fe - >3,000 reactions include these targets with these elements. This is also reflected in the list of the ten most frequent target materials appearing in the dataset (Table 3). The next-most prevalent targets are materials with Li, Ca, Nb, Mn, Bi - 2,000–3,000 reactions with these elements in targets. The least common elements are Au, Pt, Os, Be - <13 reactions in the dataset contain these elements. The rare and radioactive elements such as francium, radium, technetium or promethium are not presented in the target materials of the dataset.

We also examined the co-occurrence of chemical elements and the most typical counter-ions in precursor materials, and determined the average firing temperature used with each of these precursors. Here, we operationally define the firing temperature as the temperature used during the last heating step in the sequence of synthesis operations. The results are shown in Fig. 2 as bar-graphs for each element. The color of the bar correspond to a specific counter-ion. The pure element as precursor is shown in magenta. The length of the bar denotes the average firing temperature.

With this representation, we observe that the dataset accurately depicts known aspects of solid-state chemistry. For example, alkali and transition metal cations are often introduced into a reaction via a variety of precursors, including binary oxides, nitrides, sulfides, etc; or simple salts such as carbonates, phosphates, and nitrates. At the same time, some of the cations in precursor compounds can be found only in the form of oxides or pure elements (e.g. Be, Sc, Hf, Ru, Os, Rh, Pb, Nb, Pt, Au, …).

In solid-state synthesis, the counter-ion governs the melting or decomposition temperature of the precursor and may determine when the precursor becomes active during synthesis. The distribution of firing temperatures in Fig. 2 agrees very well with this statement and illustrates how different precursors are used in different temperature regimes during solid-state synthesis. For example, the blue bars have in general larger length (high average temperature) than red ones, because transition metal borides, carbides and nitrides often have higher reaction temperatures than their corresponding oxides, due to the refractory nature of their precursors. On the other hand, the green bars are relatively shorter (lower average firing temperature) than red ones, because, compared to oxides and complex oxide anions (carbonates, phosphates, etc), synthesis with hydroxides, oxalates, and acetates facilitate lower temperature reactions as they are often homogeneously mixed by precipitation from solution. This data-driven temperature analysis is based on precursor, and we acknowledge that reaction temperatures also depend on the thermal stability and reactivity of the target compounds. Nonetheless, the figure provides a semi-quantitative starting point for the researchers: If a target material decomposes at relatively low temperature, it may be better to choose a precursor that tends to become active at lower temperature.

**Fig. 3:**
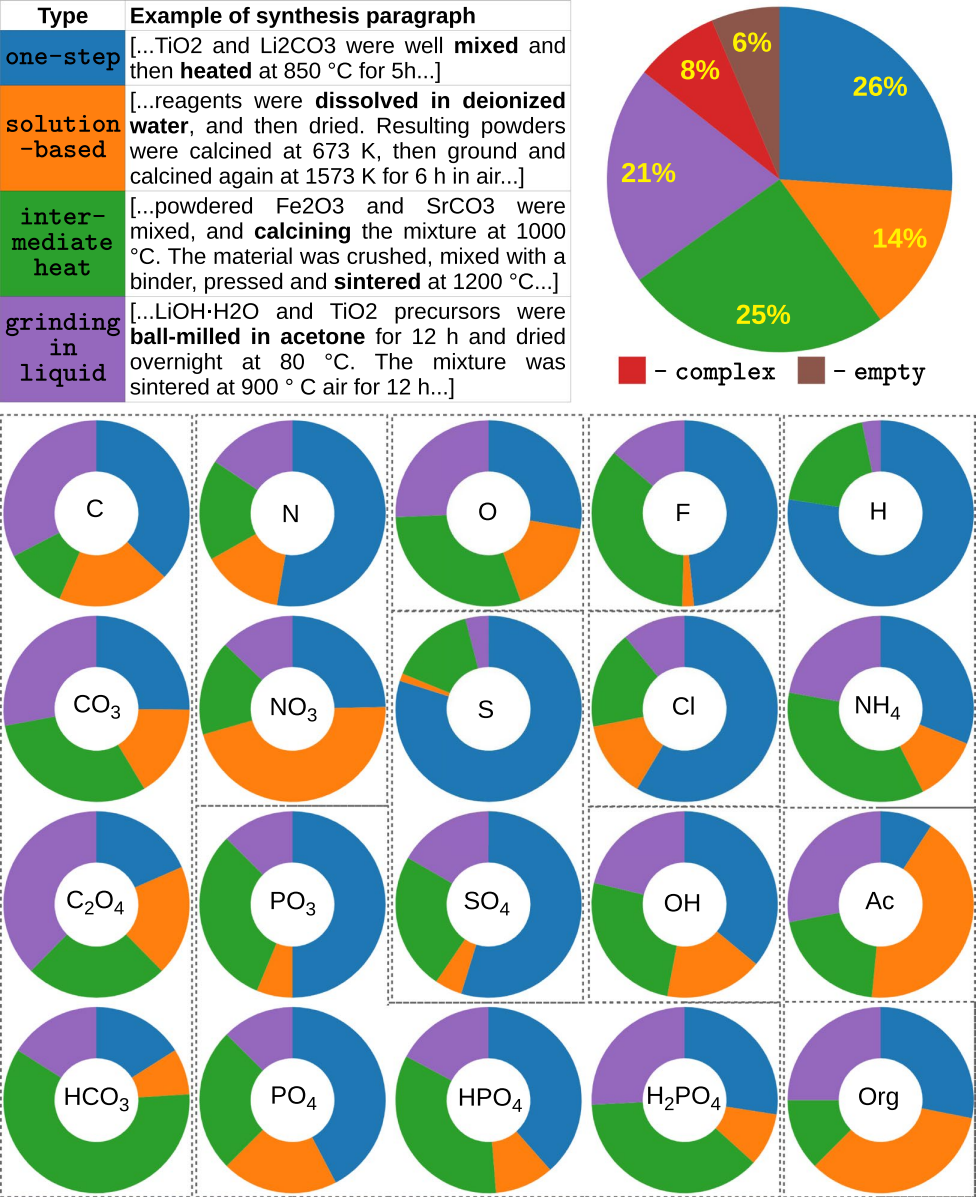
Correspondence between choice of synthesis route and precursors counter-ions. The top table gives an example of the four synthesis types defined: one-step synthesis, solution-based, synthesis with intermediate heating steps, synthesis including grinding of precursors in liquid media. The pie-charts on the right displays the fraction of each synthesis route in the dataset. The donuts-like charts represent the fractions of the four synthesis routes (given in table) for each counter-ions used in precursors. “Ac” stands for acetate radical CH_3_COO^−^ in the compound formula. “Org” stands for organic radical (–CH–) in the compound formula.

In order to demonstrate the diversity of synthesis routes represented in the dataset, we sorted the sequence of synthesis steps according to the following pre-defined patterns (table in Fig. 3):

- *one-step synthesis* consists of only solid mixing/grinding operations and at most one heating steps (final firing) without regrinding,
- *synthesis with grinding in a liquid media* to homogenize (without dissolution) the starting materials in any liquid media,
- *solution-based synthesis* contains any type of dissolution of starting materials in solvent,
- *synthesis with intermediate heat* has one or more heating steps (not including drying after mixing with liquid part) before final firing of the materials.

First, we found that different synthesis types are represented in the database almost evenly (top pie-chart in Fig. 3): 26% of materials are synthesized in one-step, 25% of the syntheses routes are done with intermediate heating step(s) before finial firing, 21% of the syntheses contain grinding (homogenizing) in liquid, and 14% require dissolving of precursors in solvent. The rest of the recipes (14%) either do not contain any detailed synthesis procedure (6%), or the pathway is more complex (8%).

Since the choice of counter-ion used in a precursor often depends strongly on the synthesis method, we surveyed which type of synthesis is common for a specific ion in precursor. We queried a subset of reactions which include the given counter-ion in a precursor compound, and calculated the fraction of each synthesis type in this subset. The resulting pie-charts are shown in Fig. 3. The emerging picture is consistent with known aspects of solid-state synthesis. For example, in the precipitation of solids during synthesis, the precursor is dissolved in the solution. As shown in Fig. 3, the solution-based synthesis (orange fraction) often uses soluble precursors with nitrates, acetates, and organic (CH-containing) radicals. Some counter-ions are more amenable to one-step synthesis than others, for example, chlorides, sulfides, and hydrides do not require much additional processing. On the other hand, relatively stable precursors such as oxides and carbonates are processed in a variety of ways, often requiring intermediate heating and grinding. This is probably due to the common formation of reaction impurities and non-equilibrium intermediates during reaction sequences^45,46^.

The extraction pipeline we developed allows for automatic processing of scientific paragraphs and identifying key information about solid-state synthesis from there. However, the pipeline still suffers from some issues with the text mining. First, most of the errors down the pipeline are introduced due to incorrect tokenization of the paragraphs and sentences. Although the ChemDataExtractor^22^ tokenizer significantly outperforms other NLP packages on chemistry-related texts, it still fails to correctly process large mixtures and solid solutions formulas as well as chemical names consisting of multiple words. We attribute this issue to the fact that ChemDataExtractor was trained on organic chemical entities, and using it for the recognition of inorganic tokens requires modification of the algorithms. Secondly, no established template or pattern exists for describing synthesis procedure which results in significant amount of ambiguity and difficulty when a synthesis method is interpreted even by an expert^47^. This requires development of more advanced text extraction models considering the features of scientific text flow. Third, although the dataset was generated from the paragraphs describing solid-state synthesis (as defined by a classification algorithm), it also contains reactions for solution-based precursors synthesis, such as sol-gel (Fig. 3). However, these entries mostly dropped out later in the pipeline, because the majority of them uses organic precursors with complex radicals, and balancing such chemical equations becomes complicated. Lastly, we found that most of the materials studied and synthesized after 2000’s are often modified (e.g. doped, elements substituted) compounds, mixtures, glasses or solid solutions. Parsing such materials into composition and building balanced reaction equations is not straightforward. For some compounds with doped and substituted elements, we included the information about modifying elements and corresponding precursors in the reaction string (see Methods). One of the ways to reconstruct reactions for mixtures, solid solutions, alloys, etc. is to split the entire material into compounds and match them with the corresponding precursors. Rather than fully resolve it, we choose to setup a flexible data structure which allows for its further development by the user.

## Usage Notes

The dataset is provided in a single file in JSON format. It can be read using all major programming languages, including Python, Matlab, R, Wolfram Mathematica. No specific technical setup is required as a dependency.

**Fig. 4:**
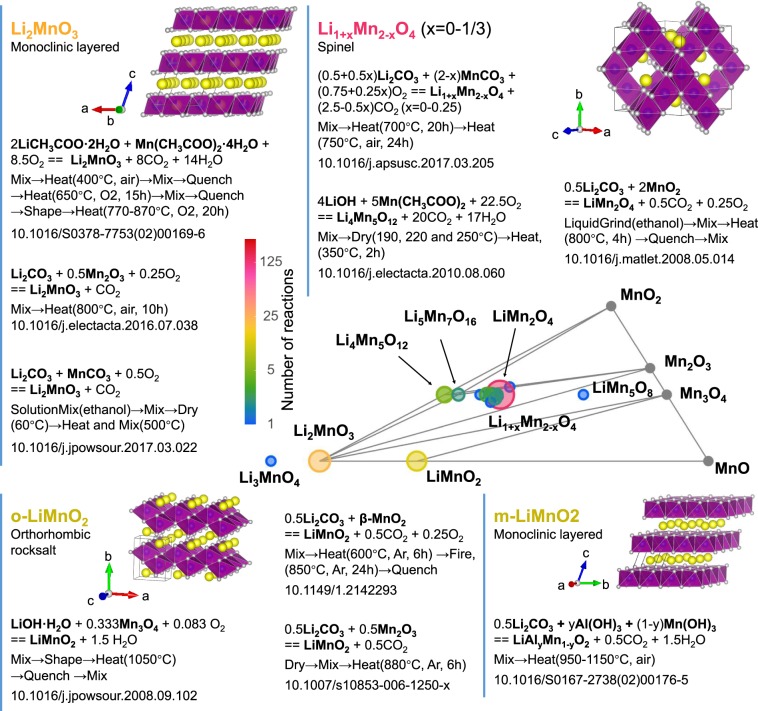
Graphical representation of dataset entries queried for the Li-Mn-O system. Examples of the subset entries: target LMO material, synthesis reaction and route. The DOIs are provided for reference. The triangle shows the distribution of the LMO materials on the phase diagram. The circles size and color are scaled according to the number of reaction in the dataset with the given target material.

The dataset can be easily queried by target and precursor compound(s), their compositions and Materials Project IDs, type of operations used in synthesis, conditions and reaction. As an example, Fig. 4 illustrates the utility of the dataset in conducting rapid literature review of different synthesis procedures within a single chemical space. It displays the result of a query for reactions to a target with Li, Mn and O in the composition. This example provides a birds-eye perspective of the various solid-state synthesis routes to target LMO compounds in this space using the dataset. The generated subset can be further queried by precursors types, as well as by type of heating/mixing conditions.

Although the dataset is provided as a static snapshot^44^, we plan to update it on a regular basis. The updates will be posted at the github repository at https://github.com/CederGroupHub/text-mined-synthesis_public.

## Data Availability

The scripts utilized to classify paragraphs and extract recipes as well as to perform the data analysis are home-written codes which are publicly available at the github repository https://github.com/CederGroupHub/text-mined-synthesis_public with acknowledgement of the current paper. The underlying machine-learning libraries used in this project are all open-source: *Tensorflow* (www.tensorflow.org), *Keras* (keras.io), *SpaCy* (spacy.io)^42^, *gensim* (radimrehurek.com)^41^ and *scikit-learn* (scikit-learn.org)^48^ *ChemDataExtractor* (chemdataextractor.org)^22^.
