# Author Correction: Text-mined dataset of inorganic materials synthesis recipes

**DOI:** 10.1038/s41597-019-0297-x

**Published:** 2019-11-15

**Authors:** Olga Kononova, Haoyan Huo, Tanjin He, Ziqin Rong, Tiago Botari, Wenhao Sun, Vahe Tshitoyan, Gerbrand Ceder

**Affiliations:** 1 Department of Materials Science and Engineering, University of California, Berkeley, CA 94720 USA; 2 Materials Sciences Division, Lawrence Berkeley National Laboratory, Berkeley, CA 94720 USA; 3 Present Address: Institute of Mathematics and Computer Sciences, University of São Paulo, São Carlos, SP Brazil; 4 Present Address: Google LLC, Mountain View, CA USA

Correction to: *Scientific Data* 10.1038/s41597-019-0224-1, published online 15 October 2019

Following publication, it was found that an incorrect grant number was attributed to the National Science Foundation in the Acknowledgements section of this Data Descriptor. This has been corrected in both the HTML and PDF versions.

